# Longitudinal assessment of anti-PGL-I serology in contacts of leprosy patients in Bangladesh

**DOI:** 10.1371/journal.pntd.0006083

**Published:** 2017-12-11

**Authors:** Renate A. Richardus, Konrad van der Zwet, Anouk van Hooij, Louis Wilson, Linda Oskam, Roel Faber, Susan J. F. van den Eeden, David Pahan, Khorshed Alam, Jan Hendrik Richardus, Annemieke Geluk

**Affiliations:** 1 Department of Infectious Diseases Leiden University Medical Center, Leiden, The Netherlands; 2 Department of Public Health, Erasmus MC, University Medical Center Rotterdam, Rotterdam, The Netherlands; 3 KIT Biomedical Research, Royal Tropical Institute, Amsterdam, The Netherlands; 4 Rural Health Program, The Leprosy Mission International Bangladesh, Nilphamari, Bangladesh; Institut Pasteur, FRANCE

## Abstract

**Background:**

Despite elimination efforts, the number of *Mycobacterium leprae* (*M*. *leprae*) infected individuals who develop leprosy, is still substantial. Solid evidence exists that individuals living in close proximity to patients are at increased risk to develop leprosy. Early diagnosis of leprosy in endemic areas requires field-friendly tests that identify individuals at risk of developing the disease before clinical manifestation. Such assays will simultaneously contribute to reduction of current diagnostic delay as well as transmission. Antibody (Ab) levels directed against the *M*.*leprae*-specific phenolic glycolipid I (PGL-I) represents a surrogate marker for bacterial load. However, it is insufficiently defined whether anti-PGL-I antibodies can be utilized as prognostic biomarkers for disease in contacts. Particularly, in Bangladesh, where paucibacillary (PB) patients form the majority of leprosy cases, anti-PGL-I serology is an inadequate method for leprosy screening in contacts as a directive for prophylactic treatment.

**Methods:**

Between 2002 and 2009, fingerstick blood from leprosy patients’ contacts without clinical signs of disease from a field-trial in Bangladesh was collected on filter paper at three time points covering six years of follow-up per person. Analysis of anti-PGL-I Ab levels for 25 contacts who developed leprosy during follow-up and 199 contacts who were not diagnosed with leprosy, was performed by ELISA after elution of bloodspots from filter paper.

**Results:**

Anti-PGL-I Ab levels at intake did not significantly differ between contacts who developed leprosy during the study and those who remained free of disease. Moreover, anti-PGL-I serology was not prognostic in this population as no significant correlation was identified between anti-PGL-I Ab levels at intake and the onset of leprosy.

**Conclusion:**

In this highly endemic population in Bangladesh, no association was observed between anti-PGL-I Ab levels and onset of disease, urging the need for an extended, more specific biomarker signature for early detection of leprosy in this area.

**Trial registration:**

ClinicalTrials.gov ISRCTN61223447

## Author summary

Leprosy is an infectious disease caused by the bacterium *Mycobacterium leprae*, which causes skin and nerve damage. Despite worldwide efforts to eliminate leprosy, the number of infected individuals who develop leprosy, is still substantial. Household contacts of new leprosy patients are especially at risk. Early diagnosis of leprosy is key in preventing lifelong handicaps as well as transmission. This requires field-friendly tests that identify individuals at risk of developing the disease before they develop clinical symptoms so that they can receive (prophylactic) treatment. Measuring antibody levels directed against the *M*.*leprae*-specific phenolic glycolipid I (PGL-I) provides an indication of the bacterial load. To identify whether anti-PGL-I Ab levels correlate with the development of leprosy in contacts of newly diagnosed leprosy cases, we analyzed these levels in 25 contacts who developed leprosy during 6 years of follow-up and 199 contacts who were not diagnosed with leprosy at 3 time points in 6 years. This study showed that anti-PGL-I Ab levels at intake did not significantly differ between contacts who developed leprosy during the study and those who remained free of disease. Therefore, anti-PGL-I Ab levels alone do not represent a practical tool for prediction of which household contacts will develop leprosy in an endemic area such as Bangladesh, with high levels of patients with paucibacillary leprosy. This stresses the need for a diagnostic test composed of a biomarker signature consisting of multiple biomarkers.

## Introduction

Leprosy is an infectious disease caused by *Mycobacterium leprae* (*M*. *leprae*), which causes damage to the skin and peripheral nerves[1]. The highest numbers of new leprosy cases are detected in India (127,326 in 2015), Brazil (26,395 in 2015) and Indonesia (17,202 in 2015)[2]. Bangladesh also has highly endemic areas, with a number of new cases of above 3,000 per year[2]. Although leprosy prevalence has decreased tremendously along with the widespread availability of multidrug therapy (MDT) in endemic areas, detection of new cases worldwide has shown only a modest decline in the last five years, and has stabilized in some countries[3]. In Bangladesh, the number of new cases was 3,976 in 2015, compared to 3,848 new cases in 2010[2]. Indirect evidence indicates that worldwide millions of unreported cases linger undetected as a gradual result of a decline in leprosy control activities after the disease was declared eliminated[1]. The continued transmission is probably largely due to *M*. *leprae* infected individuals, carrying substantial numbers of bacteria but (yet) lacking clinical symptoms. Thus, early detection and subsequent (prophylactic) treatment of asymptomatically infected individuals as well as subclinical disease is essential to reduce transmission.

Diagnosis of leprosy is still largely dependent on clinical signs and symptoms and detection of acid fast bacteria. However, user friendly lateral flow assays provide new possibilities for rapid diagnosis of leprosy patients in early stages of the disease or of *M*. *leprae* infected individuals without any symptoms[4, 5]. Such assays are likely to contribute to reduction of current diagnostic delay in endemic areas and also aid classification of leprosy disease, allowing appropriate treatment. Currently, there is no specific and sensitive test available that can detect asymptomatic *M*. *leprae* infection or predict progression to clinical disease[6]. In view of the long incubation time of leprosy (typically 3–5 years) as well as its low incidence, identification of predictive biomarkers requires longitudinal monitoring of *M*. *leprae*-specific immunity in those at risk of developing disease. Therefore, investments in large-scale longitudinal follow-up studies, allowing intra-individual comparison of immune profiles in leprosy patients’ contacts, is essential to evaluate which markers correlate with progression to disease and may be used as predictive biomarkers.

*M*. *leprae* phenolic glycolipid I (PGL-I) is an extensively studied antigen on the outer surface of the mycobacterium[7]. The existence of high levels of IgM antibodies to PGL-I[5–7], has allowed the development of several tests that were investigated broadly for diagnostic purposes[7–10]. Although useful for identifying multibacillary (MB) leprosy patients, anti-PGL-I antibody (Ab) titers have little value in detecting PB leprosy patients, since the latter develop cellular rather than humoral immunity and therefore often lack antibodies to PGL-I^5^.

In a previously conducted cluster randomized controlled trial, designated the COLEP study, the effect of single dose rifampicin versus placebo in preventing leprosy in close contacts of newly diagnosed leprosy patients was studied between 2002 and 2009 in a leprosy endemic area in the Northwest of Bangladesh[11, 12]. To investigate whether anti-PGL-I Ab seropositivity can be used as a predictive biomarker for progression to leprosy in contacts, the current study compared anti-PGL-I Ab levels of the prospective cohort at intake and at three time points covering six years of follow-up per contact.

## Methods

### Study participants

Contacts of leprosy patients were voluntarily recruited as part of the COLEP study (a cluster randomized controlled trial) in 2002 and 2003 in the districts Rangpur and Nilphamari in the northwest of Bangladesh, which is a leprosy endemic area[11, 12]. Eligible participants (patients and contacts) were informed verbally about the study and invited to participate. Written consent was obtained from all participants at recruitment or from the parent or guardian of under 18s. Contacts were followed prospectively from 2002/2003 to 2008/2009 for the development of leprosy. Blood samples were collected by spotting on Whatman filter paper (Sigma) and subsequently stored at -80°C. Blood samples were collected at 4 time points: recruitment into the study, follow-up 1 (FU1; two years after intake), follow-up 2 (FU2; four years after intake) and follow-up 3 (FU3; six years after intake)[12]. Leprosy was diagnosed when at least one of the following signs was present: one or more skin lesions with sensory loss, thickened peripheral nerves, or a positive skin smear result for acid-fast bacilli. Patients with negative smear results and no more than five skin lesions were classified as PB leprosy, and those with a positive smear or more than five skin lesions as MB leprosy[12]. Clinical and demographic data was collected in the COLEP study database[11].

### Test group selection

A random sample was taken from 28,092 contacts of leprosy patients recruited within the COLEP study[11]. A total of 239 contacts developed leprosy within the six years of follow-up. 25 contacts were included into this sub-study who were diagnosed with leprosy at either FU1, FU2 or FU3 and for whom filter papers of at least three different time points were available. Out of the contacts who did not develop leprosy, 199 were randomly included using the RAND formula (Excel 2010), aiming for an equal ratio of three age groups (0–14, 15–29, and 30+ years).

The COLEP study represents a unprecedented field trial for leprosy, because it includes valuable longitudinal analysis of contacts and thus is uniquely suited to identify the predictive value of biomarkers. However, the COLEP study did not collect blood samples from contacts as the only samples collected was blood on filter paper. Therefore, this limited biomarker analysis to anti-PGL-I Ab only.

### Leprosy prevalence

In this part of the country, the new case detection rate of leprosy was 3.21 per 10,000 in 2002 (DBLM Annual Report 2002). In these cases leprosy was diagnosed by active and passive case detection. In 2002 and 2003 random samples from the general population were taken to calculate the prevalence of previously undiagnosed leprosy (PPUL). In the contact group of the COLEP study, the PPUL rate was 73/10,000, compared to 15.1/10,000 in the samples taken from the general population. These cases were found by active door-to-door screening[13].

### Synthetic PGL-I

Disaccharide epitope (3,6-di-O-methyl-β-D-glucopyranosyl(1→4)2,3-di-O-methylrhamnopyranoside) of *M*. *leprae* specific native PGL-I glycolipid was synthesized and coupled to human serum albumin (synthetic PGL-I; designated ND-O-HSA). This was generated with support from the NIH/NIAID Leprosy Contract N01-AI-25469 and obtained through the Biodefense and Emerging Infections Research Resources Repository (http://www.beiresources.org/TBVTRMResearchMaterials/tabid/1431/Default.aspx).

### PGL-I ELISA

Antibodies (IgM, IgG, IgA) against *M*. *leprae* PGL-I were detected as described previously[5, 14, 15]. ND-O-HSA was coated onto high-affinity polystyrene Immulon 4HBX 96-well Nunc ELISA plates (Thermo Scientific, Rochester, NY) using 500 ng per well in 50 μl of 0.1M sodium carbonate/bicarbonate pH 9.6 (i.e. coating buffer) at 4°C overnight. Unbound antigen was removed by washing six times with PBS (phosphate buffered saline) with 0,05% Tween 20 (wash buffer). The wells were blocked with PBS containing 1% BSA (bovine serum albumin) (Roche Diagnostics, Germany) for 1 hour at room temperature (RT). Bloodspots were punched from filter papers. Three punches (2 mm each) per individual were added to 100 μl PBST (PBS/0,1% Tween20) and incubated at 4°C in 24 wells plates. After overnight incubation, 50 μl PBST/NRS (PBST + 10% normal rabbit serum) was added to each well and the plates were shaken gently for 1 hour at RT. The eluate was added to the ELISA plates (50 μl/ well) and incubated for 2 hours at RT. After incubating with the primary antibody, the wells were washed six times with PBS with 0.05% Tween 20 (wash buffer), followed by the addition of 50 μl of a 1:8,000 dilution of the secondary antibody anti-human (Dako P0212) for two hours. Following washing the wells with the wash buffer six times, 50 μl of *p*-nitrophenylphosphate substrate (Kirkegaard and Perry Labs, Gaithersburg, MD) was added. Antibodies (IgM, IgG, IgA) against *M*. *leprae* PGL-I were detected as previously described[14]. Absorbance was determined at a wavelength of 450 nm. Samples with an optical density at 450 nm (OD_450_), after correction for background OD above 0.150, were considered positive. This threshold was determined by a threefold multiplication of an average EC value.

As quality control, anti-PGL-I IgM levels were determined for 10 Dutch leprosy patients by ELISA using serum as well as blood spots on filter paper: Although IgM levels were higher for 9 individuals in sera, all seropositive individuals were also positive using blood spots and OD_450_ values correlated well (R^2^ = 0,80).

### Statistical analyses

Multivariable logistic regression was used to calculate adjusted odds ratios for the level of ant-PGL-I Ab levels at intake, and corrected for age and sex. A p-value ≤ 0.05 was used as a cut-off for statistical significance. To investigate the association of changes in anti-PLG-I Ab from baseline to the time of development of leprosy, generalized linear mixed models were used. The dependent variable was the development of leprosy at a time point and the differences in anti-PLG-I Ab levels from baseline were included as independent variables. To adjust for the correlation between intra-individual measurements we included a random intercept for each subject. The difference between the anti-PGL-I Ab levels between contacts of MB or PB index patients was calculated using a t-test comparing averages. All analyses were performed in R version 3.2.0 (R, Vienna, Austria; https://www.R-project.org).

## Results

From the 28,092 contacts of leprosy patients recruited within the COLEP study[11], 239 contacts developed leprosy within the six years of follow-up. For 25 contacts who were diagnosed with leprosy during follow-up and 199 contacts who remained free of leprosy, good quality filter paper was available for at least three different time points during follow-up.

Characteristics of the study populations are shown in Table 1 and Table 2. Of the 25 contacts who developed leprosy, 10 contacts developed leprosy at 2 years after intake (FU1), 7 contacts at 4 years after intake (FU2) and 8 contacts at 6 years after intake (FU3). Four contacts (16%) developed MB leprosy and 21 (84%) developed PB leprosy. This is the same proportion of MB versus PB as in the total group of new leprosy cases diagnosed within the COLEP study[12] 4 years after intake (24 MB contacts versus 126 PB contacts; 16% versus 84%). The group was evenly distributed for sex (M/F = 1.17:1) and age categories. For 10 contacts the index patient had MB leprosy, whereas for 15 contacts this was PB leprosy.

**Table 1:** Contact characteristics.

	Contacts who developed leprosy (n = 25)	Contacts who did not develop leprosy (n = 199)
Male	14	91
Female	11	108
Age (1–15 years)	8	65
Age (16–30 years)	9	54
Age (31+ years)	8	80
Leprosy at FU1	11	0
Leprosy at FU2	7	0
Leprosy at FU3	7	0
MB	4	0
PB	21	0

Characteristics of the contacts of new leprosy patients that either developed (n = 25) or did not (n = 199) develop leprosy. Sex, age group, time point of leprosy development (FU1: two years after intake; FU2: four years after intake; FU3: six years after intake) and type of leprosy developed (MB: multibacillary leprosy; PB: paucibacillary leprosy) were specified.

**Table 2:** Ridley Jopling classification of contacts with leprosy.

Ridley-Jopling Classification	Contacts with PB leprosy (n = 21)	Contacts with MB leprosy (n = 4)
I	1	0
TT	3	0
BT	17	4

Ridley-Jopling classification of contacts (n = 25) of new leprosy patients who developed leprosy at follow-up. Contacts developed either paucibacillary leprosy (PB): indeterminate (I), tuberculoid (TT) or borderline tuberculoid (BT) leprosy; or multibacillary leprosy (MB): borderline tuberculoid (BT) leprosy.

**Fig 1:**
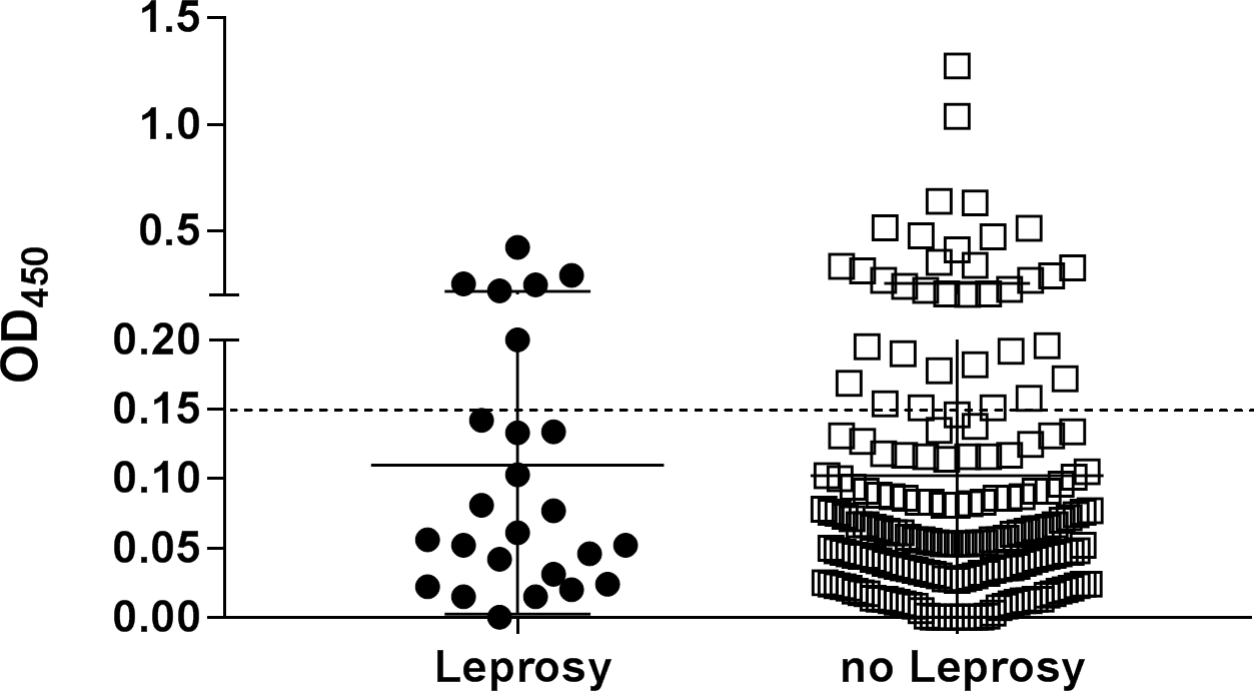
Cross-sectional analysis of anti-PGL-I Ig antibody levels at intake. Anti-PGL-I antibodies at intake for contacts of leprosy patients who developed leprosy during the study (black circle; n = 25) and contacts who remained free of leprosy disease (white box; n = 198) were detected by ELISA using natural disaccharide of PGL-I linked to HSA (ND-O-HSA). Optical density readings were performed at 450nm (OD_450_) and corrected for background levels. Median values per group are indicated by horizontal lines. The cut-off for positivity is indicated by the dashed horizontal line.

The anti-PGL-I Ab levels at intake were compared between the two groups of contacts (Fig 1). In the group of contacts who developed leprosy, the average anti-PGL-I Ab titer at intake was 0.11, and varied between zero and 0.424. 6 of these 25 (24%) contacts who developed leprosy had a positive anti-PGL-I Ab level of >0.150 at intake. In the group who did not develop leprosy, the average anti-PGL-I Ab titer was 0.10 and varied between zero and 1.275. 35 out of 199 (17.6%) contacts who did not develop leprosy had a positive anti-PGL-I Ab level of >0.15 at intake. No significant association was observed for the anti-PGL-I Ab levels at baseline (OR: 1.01 (0.78, 1.31), 95% CI p = 0.94) between the two groups.

**Fig 2:**
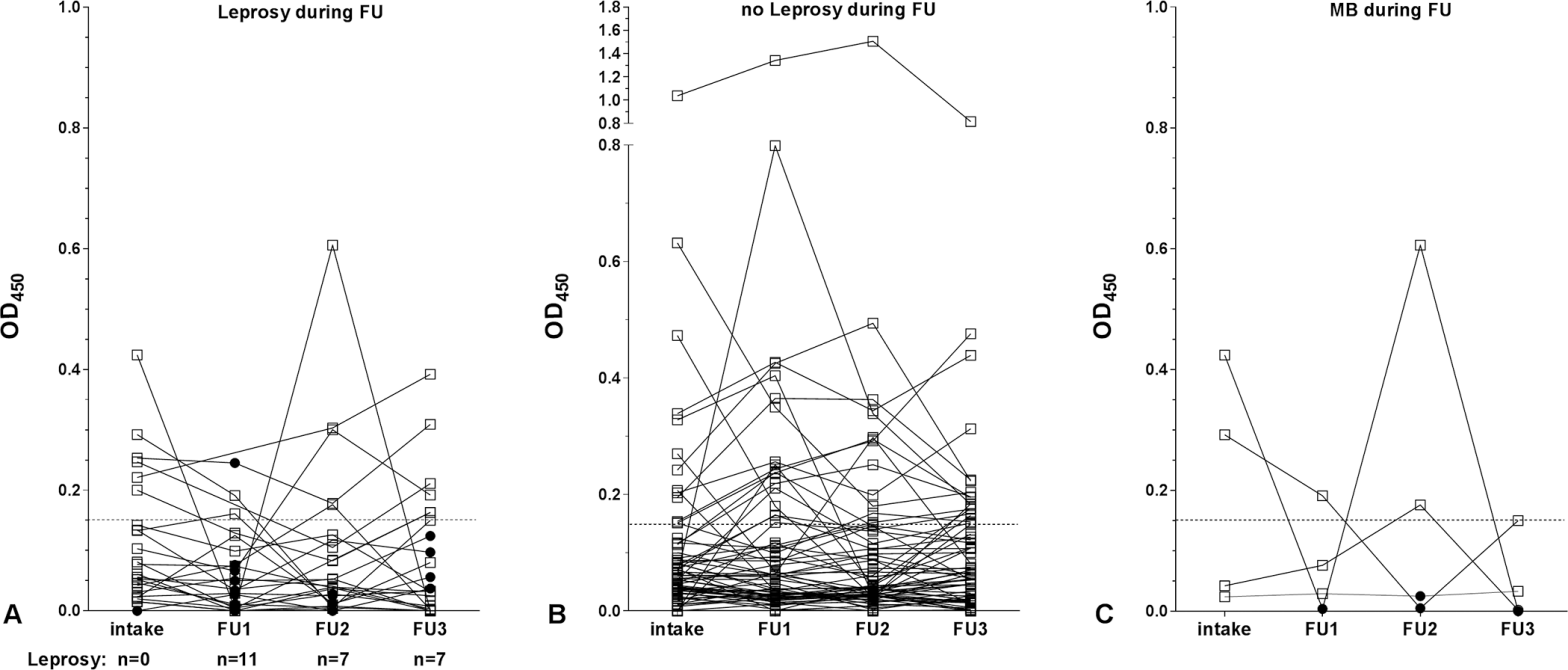
Longitudinal analysis of anti-PGL-I Ig antibody levels. Anti-PGL-I antibodies for all contacts of leprosy patients who developed leprosy during the study (**A**; n = 25) and contacts who remained free of leprosy disease (**B**; n = 199) and 4 contacts who developed MB leprosy (**C**; n = 4) were determined by ELISA using natural disaccharide of PGL-I linked to HSA (ND-O-HSA). Sera were tested at three follow-up time points; FU1: 2 years after intake, FU2: 4 years after intake, FU3: 6 years after intake. Optical density readings were performed at 450nm (OD_450_) and corrected for background levels. Anti-PGL-I Ab levels at the time point of leprosy diagnosis are indicated with black circles (**A** and **C**). The cut-off for positivity is indicated by the dashed horizontal lines.

To further analyze the longitudinal pattern of PGL-I serology in contacts, the anti-PGL-I Ab levels are depicted at different follow-up times, comparing the titers of contacts developing leprosy (Fig 2A) to the titers of contacts without leprosy (Fig 2B). The difference between anti-PGL-I Ab level at diagnosis was compared to the anti-PGL-I Ab level at intake. This difference was minus 0.047, indicating that the level of anti-PGL-I Ab titer was lower at time of diagnosis compared to time of intake. Next we calculated the difference between anti-PGL-I Ab titer at various time points of follow-up to the anti-PGL-I Ab level at intake of the contacts who did not develop leprosy using a generalized linear mixed model analysis. Thus, for all contacts who did not develop leprosy, we compared the anti-PGL-I Ab level at FU1 to intake, the level of FU2 to intake and the level at FU3 to intake. If a contact developed leprosy at FU2 (or FU3), we also included the difference between anti-PGL-I Ab titer at FU1 (and FU2) and intake into the group of contacts who did not develop leprosy. Differences in anti-PGL-I Ab levels had no significant association with the development of leprosy at either of the three follow-up time points (OR: 0.62 (0.15, 2.62), p = 0.52). Thus changes in anti-PLG-I Ab levels are not predictive of disease progression in contacts of new leprosy patients in Bangladesh. Since MB patients harbor a higher quantity of bacteria than PB, we separately considered the longitudinal pattern of the anti-PGL-I Ab levels in the four contacts who developed MB leprosy (Fig 2C). The mean OD450 at the time of diagnosis for both MB/BT and PB patients was below threshold for positive (< 0.15). Also, no increase in anti-PGL-I Ab levels was observed at the moment of leprosy diagnosis; actually, anti-PGL-I Ab levels were often even lower at diagnosis time compared to intake. The findings indicate that not only for newly diagnosed PB, but also for MB patients, anti-PGL-I Ab levels do not represent a practical tool for prediction of leprosy.

## Discussion

Although several studies described that positive anti-PGL-I Ab titers in household contacts of leprosy patients were related to a higher risk of developing leprosy[16–19], reports also indicated that more than half of the individuals with antibodies against PGL-I will never develop leprosy[16, 17]. Besides, diagnosis based only on seropositivity for anti-PGL-I Abs would leave more than half of the new leprosy cases undetected[16, 18, 19]. To study the value of anti PGL-I Ab as a predictor of leprosy in those at risk of developing leprosy in a highly endemic area, we here analyzed the anti PGL-I Ab levels in the blood of 224 contacts of leprosy patients in the Northwest part of Bangladesh. However, no association was found between anti-PGL-I Ab levels and onset of disease in this population.

As part of a variety of studies investigating the use of serology for prediction of leprosy in those at risk of developing disease, a study in the state of Minas Gerais, Brazil[18] suggested that anti-PGL-I serology in household contacts of leprosy patients can be used to identify leprosy at a preclinical stage. This study identified more contacts with suspected leprosy in the group with positive anti-PGL-I levels (9.62%) than in the test-negative group (1.76%). However, out of the 52 contacts with positive anti-PGL-I serology, only 5 had leprosy. The anti-PGL-I seropositivity was higher in those contacts exposed to patients with MB leprosy than PB leprosy, which is probably due to the higher bacterial load in MB patients and therefore higher exposure rates of their contacts.

In another Brazilian study[17], performed in Rio de Janeiro, leprosy diagnosis had a strong association with anti-PGL-I seropositivity at intake. A significantly higher proportion of healthy contacts with anti-PGL-I Abs (5.6%) developed leprosy during the follow-up period compared with those without (2.3%). Anti-PGL-I seropositive contacts had a 3.2-fold higher risk of developing leprosy compared with seronegative contacts.

A third study performed in Cebu (the Philippines)[16] showed that household contacts of MB leprosy patients with anti-PGL-I Abs have a 7.65-fold-higher risk of developing leprosy in the six years of active surveillance than seronegative contacts. It is noteworthy that out of the 27 contacts developing leprosy, 13 remained seronegative, indicating that half of the new leprosy cases would not be detected when solely anti-PGL-I serology would be used as a predictive diagnostic tool. This particularly applies to PB cases, as all of the 10 newly diagnosed MB patients were or became seropositive. On the other hand, 85 out of the 99 anti-PGL-I Ab positive contacts never developed leprosy, implying a false positivity rate of 86% when using anti-PGL-I serology as a predictive marker for leprosy.

Barretto *et al*.[20] showed that the odds of seropositive versus seronegative school children developing leprosy within two years is 2.7 times higher in an hyperendemic region in the Amazones of Brazil (State of Pará). Thus, this would indicate a > 90% probability of detecting at least one new case among 10 seropositive individuals in 2 years. On the other hand, 5 of 11 new cases found amongst school children in these high-risk areas in Brazil tested negative for anti-PGL-I Abs. Furthermore, no significant difference between the median anti-PGL-I Ab titer of new cases and of healthy school children was observed. Of note is that a significant increase in the anti-PGL-I IgM titers was found at the time of diagnosis compared to intake. The group that did not develop leprosy also demonstrated an increase in their average antibody titers, although the most significant increase was observed in the group that developed disease. These findings in Brazil stand in contrast to our current study in Bangladesh, in which hardly any difference or even a slight decrease in the anti-PGL-I Ab levels was observed in the contacts who developed leprosy.

A recent meta-analysis among household contacts of new leprosy patients in French Polynesia, Zaire, Papua New Guinean, Venezuela, Brazil, India and Philippines[19] shows that the risk of developing leprosy is about three times higher in those who are positive for anti-PGL-I Abs compared to the seronegative group, with the odds ratio varying from 2.72 to 3.53. However, the sensitivity of anti-PGL-I Ab tests as predictor of the development of clinical leprosy was found to be lower than 50% in all studies. Thus, selecting contacts with anti-PGL-I antibodies for prophylaxis, although possibly beneficial for reduction of transmission, would only prevent less than half of the leprosy cases among contacts. Our findings in contacts in Bangladesh are in line with those of the meta-analysis by Penna *et al.[19]* as well as the other studies discussed above, since development of leprosy was not associated with the level of anti-PGL-I seropositivity at intake, clearly indicating that also in Bangladesh anti-PGL-I Ab tests lack the ability to early diagnose leprosy amongst leprosy contacts[21–23], if used as a stand alone tool.

Most of the leprosy patients’ contacts in our study developed PB leprosy (21 out of 25), which offers an explanation for the lack of increase of anti-PGL-I titers at leprosy diagnosis. Importantly, in Bangladesh, the percentage of PB cases amongst new leprosy cases is generally higher than in other countries in Asia, especially southeast Asia where predominantly MB patients are found[2]. This phenomenon is probably due to a combination of genetic factors as well as early case detection. Bangladesh is a high endemic area with a high rate of active case-finding, which leads to a lot of PB cases being found. In contrast, low endemic areas with little active case-finding have higher numbers of MB cases, since PB is often self-healing. In our study, only four household contacts developed MB leprosy (out of the 25 total number of new leprosy patients). PB leprosy in general is characterized by low levels or absence of antibodies against *M*. *leprae* antigens[16], which is in line with our finding that there was no significant difference in anti-PGL-I Ab level at intake compared to leprosy diagnosis. Schuring *et al*[24] found that anti-PGL-I seropositivity was associated with bacterial index (BI). However, most contacts in our study had PB and therefore an undetectable BI. Separate evaluation of the four MB patients did not show any differential increase in anti-PGL-I Ab level in this group either. This is in line with the findings of van Hooij et al[4, 25], showing low levels of anti-PGL-I Ab in all patients, including MB. Moreover, anti-PGL-I IgM levels could not be used to discriminate PB patients or household contacts from endemic controls. In leprosy endemic countries other than Bangladesh, where MB leprosy is more prevalent, the longitudinal pattern of anti-PGL-I Ab levels could hold more diagnostic value. Besides this, anti-PGL-I antibodies can represent a useful tool for monitoring effectiveness of treatment of leprosy (reactions), since effective treatment is associated with decrease in antibody levels[26].

As a part of the COLEP trial, half of the new leprosy contacts received placebo and the other half single dose rifampicin. It can be expected that single dose rifampicin could lower the anti-PGL-I antibody level in subjects with a relatively high bacterial load. However, it is unknown how soon and to which extent the antibody titre is suppressed. Furthermore, there is also the possibility that subjects become re-infected with *M*. *leprae* due to continued exposure to an unknown source. Also, in the absence of complete ‘sterilisation’ of *M*. *leprae* in these subjects, the bacterium may start to multiply again after the effect of rifampicin has waned. So although the antibody titre may certainly have decreased due to single dose rifampicin, it is unknown whether this effect would be apparent after 2 years, at the moment of first blood sampling.

Furthermore, it is worthy to note that leprosy is a complicated disease with different immunological processes playing a role in disease progression, which in turn are affected by factors such as genetics[27], co-infections[28] as well as food-shortage[29]. The combination of these factors with the long incubation time that elapses before leprosy becomes clinically manifest, makes predicting which *M*. *leprae* exposed individuals will progress to disease complicated. For example, certain helminth-derived proteins can bias the host immune response towards an anti-inflammatory Th2 response, which may facilitate *M*. *leprae* growth or progression to MB leprosy[28]. Furthermore, a period of food shortage can reduce cell mediated immunity of individuals incubating *M*. *leprae*, causing the development of clinical disease[29].

Recent advancements in leprosy biomarker research[15] have shown that IFN-γ responses measured after stimulation with leprosy-unique antigens can be used as a measure for *M*. *leprae* exposure. In particular, the combination of humoral and cellular biomarkers increased efficiency to distinguish *M*. *leprae* infected from non-infected individuals, patients from contacts, or lepromatous from tuberculoid patients compared to serology alone[4, 15]. In view of the findings in this study as well as our previous studies on cellular biomarkers[4, 15, 26, 30], field-friendly tests using a biomarker signature would improve identification of contacts who are at risk of developing leprosy as well as asymptomatic, infected individuals who can transmit bacteria. In current longitudinal studies on biomarker identification, a new lateral flow test format is used (UCP-LFA)[4, 25], that not only allows field-use but also provides a permanent record as the luminescent signal on the LF strips does not fade. Such tests would represent a useful contribution to current pilot studies on the effectiveness of SDR as leprosy post-exposure prophylaxis (LPEP)[31], allowing more selective targeting for prophylaxis as well as preventing overtreatment.

### Conclusion

In view of the dichotomy of the leprosy spectrum in terms of immunity against *M*. *leprae*, current research is focused on identification of predictive biomarker profiles associated with early stage leprosy, consisting of multiple cellular and humoral (disease-specific) biomarkers. Early diagnosis of leprosy and subsequent appropriate multidrug therapy (MDT) will not only decrease severe nerve damage and subsequent lifelong handicaps, but also significantly contribute to further decrease of *M*. *leprae* transmission. This study shows that measurement of anti-PGL-I Abs alone is not sufficient to predict the development of clinical leprosy amongst household contacts of newly diagnosed leprosy cases in (highly) endemic area such as Bangladesh. Because of the high number of PB patients in Bangladesh, using anti-PGL-I titers as a screening test to discriminate which contacts to treat, may lead us to miss a lot of potential new cases.

### Ethics approval and consent to participate

Ethical clearance was obtained from the Ethical Review Committee of the Bangladesh Medical Research Council in Dhaka (ref. no. BMRC/ERC/2001-2004/799). All subjects were informed verbally in their own language (Bengali) about the study when they were invited to participate. Written consent was received from each adult, while a parent or guardian had to sign the consent form for children who participated in the study.

## Supporting information

S1 Checklist STROBE Statement–checklist of items that should be included in reports of observational studies. (DOCX)

## References

[1] Smith WC, van Brakel W, Gillis T, Saunderson P, Richardus JH. The missing millions: a threat to the elimination of leprosy. PLoS neglected tropical diseases. 2015;9(4):e0003658 Epub 2015/04/24. doi: 10.1371/journal.pntd.0003658 ; PubMed Central PMCID: PMCPMC4408099. 25905706 10.1371/journal.pntd.0003658 PMC4408099

[2] Global leprosy update, 2015: time for action, accountability and inclusion. Weekly epidemiological record 2016;91(35):405–20. 27592500

[3] Global leprosy update, 2014: need for early case detection. Wkly Epidemiol Rec. 2015;90(36):461–74. Epub 2015/09/08. . 26343055

[4] van Hooij A, Tjon Kon Fat EM, Richardus R, van den Eeden SJ, Wilson L, de Dood CJ, et al Quantitative lateral flow strip assays as User-Friendly Tools To Detect Biomarker Profiles For Leprosy. Scientific reports. 2016;6:34260 Epub 2016/09/30. doi: 10.1038/srep34260 ; PubMed Central PMCID: PMCPMC5041085. 27682181 10.1038/srep34260 PMC5041085

[5] Bobosha K, Tjon Kon Fat EM, van den Eeden SJ, Bekele Y, van der Ploeg-van Schip JJ, de Dood CJ, et al Field-evaluation of a new lateral flow assay for detection of cellular and humoral immunity against Mycobacterium leprae. PLoS neglected tropical diseases. 2014;8(5):e2845 Epub 2014/05/09. doi: 10.1371/journal.pntd.0002845 ; PubMed Central PMCID: PMCPMC4014418. 24810599 10.1371/journal.pntd.0002845 PMC4014418

[6] Geluk A. Challenges in immunodiagnostic tests for leprosy. Expert Opin Med Diagn. 2013;7(3):265–74. Epub 2013/03/30. doi: 10.1517/17530059.2013.786039 . 23537134 10.1517/17530059.2013.786039

[7] Spencer JS, Brennan PJ. The role of Mycobacterium leprae phenolic glycolipid I (PGL-I) in serodiagnosis and in the pathogenesis of leprosy. Lepr Rev. 2011;82(4):344–57. Epub 2012/03/24. . 22439275

[8] Spencer JS, Kim HJ, Wheat WH, Chatterjee D, Balagon MV, Cellona RV, et al Analysis of antibody responses to Mycobacterium leprae phenolic glycolipid I, lipoarabinomannan, and recombinant proteins to define disease subtype-specific antigenic profiles in leprosy. Clin Vaccine Immunol. 2011;18(2):260–7. Epub 2010/12/24. doi: 10.1128/CVI.00472-10 ; PubMed Central PMCID: PMCPMC3067349. 21177913 10.1128/CVI.00472-10 PMC3067349

[9] Geluk A, Duthie MS, Spencer JS. Postgenomic Mycobacterium leprae antigens for cellular and serological diagnosis of M. leprae exposure, infection and leprosy disease. Lepr Rev. 2011;82(4):402–21. Epub 2012/03/24. . 22439280

[10] Duthie MS, Raychaudhuri R, Tutterrow YL, Misquith A, Bowman J, Casey A, et al A rapid ELISA for the diagnosis of MB leprosy based on complementary detection of antibodies against a novel protein-glycolipid conjugate. Diagnostic microbiology and infectious disease. 2014;79(2):233–9. Epub 2014/03/29. doi: 10.1016/j.diagmicrobio.2014.02.006 . 24666703 10.1016/j.diagmicrobio.2014.02.006

[11] Feenstra SG, Pahan D, Moet FJ, Oskam L, Richardus JH. Patient-related factors predicting the effectiveness of rifampicin chemoprophylaxis in contacts: 6 year follow up of the COLEP cohort in Bangladesh. Lepr Rev. 2012;83(3):292–304. . 23356030

[12] Moet FJ, Pahan D, Oskam L, Richardus JH. Effectiveness of single dose rifampicin in preventing leprosy in close contacts of patients with newly diagnosed leprosy: cluster randomised controlled trial. BMJ. 2008;336(7647):761–4. Epub 2008/03/12. bmj.39500.885752.BE [pii] doi: 10.1136/bmj.39500.885752.BE ; PubMed Central PMCID: PMC2287265. 18332051 10.1136/bmj.39500.885752.BE PMC2287265

[13] Moet FJ, Schuring RP, Pahan D, Oskam L, Richardus JH. The prevalence of previously undiagnosed leprosy in the general population of northwest Bangladesh. PLoS Negl Trop Dis. 2008;2(2):e198 Epub 2008/02/28. doi: 10.1371/journal.pntd.0000198 ; PubMed Central PMCID: PMC2254205. 18301731 10.1371/journal.pntd.0000198 PMC2254205

[14] Khadge S, Banu S, Bobosha K, vdP-vS JJ, Goulart IM, Thapa P, et al Longitudinal immune profiles in type 1 leprosy reactions in Bangladesh, Brazil, Ethiopia and Nepal. BMC Infect Dis. 2015;15(1):477 doi: 10.1186/s12879-015-1128-0 26510990 10.1186/s12879-015-1128-0 PMC4625471

[15] Geluk A, Bobosha K, van der Ploeg-van Schip JJ, Spencer JS, Banu S, Martins MV, et al New biomarkers with relevance to leprosy diagnosis applicable in areas hyperendemic for leprosy. J Immunol. 2012;188(10):4782–91. doi: 10.4049/jimmunol.1103452 . 22504648 10.4049/jimmunol.1103452 PMC3345093

[16] Douglas JT, Cellona RV, Fajardo TT Jr., Abalos RM, Balagon MV, Klatser PR. Prospective study of serological conversion as a risk factor for development of leprosy among household contacts. Clin Diagn Lab Immunol. 2004;11(5):897–900. Epub 2004/09/11. doi: 10.1128/CDLI.11.5.897-900.2004 ; PubMed Central PMCID: PMCPMC515277. 15358649 10.1128/CDLI.11.5.897-900.2004 PMC515277

[17] Duppre NC, Camacho LA, Sales AM, Illarramendi X, Nery JA, Sampaio EP, et al Impact of PGL-I seropositivity on the protective effect of BCG vaccination among leprosy contacts: a cohort study. PLoS Negl Trop Dis. 2012;6(6):e1711 Epub 2012/06/23. doi: 10.1371/journal.pntd.0001711 ; PubMed Central PMCID: PMCPMC3378622. 22724040 10.1371/journal.pntd.0001711 PMC3378622

[18] Carvalho AP, da Conceicao Oliveira Coelho Fabri A, Correa Oliveira R, Lana FC. Factors associated with anti-phenolic glycolipid-I seropositivity among the household contacts of leprosy cases. BMC infectious diseases. 2015;15:219 Epub 2015/05/31. doi: 10.1186/s12879-015-0955-3 ; PubMed Central PMCID: PMCPMC4449587. 26024906 10.1186/s12879-015-0955-3 PMC4449587

[19] Penna ML, Penna GO, Iglesias PC, Natal S, Rodrigues LC. Anti-PGL-1 Positivity as a Risk Marker for the Development of Leprosy among Contacts of Leprosy Cases: Systematic Review and Meta-analysis. PLoS Negl Trop Dis. 2016;10(5):e0004703 Epub 2016/05/19. doi: 10.1371/journal.pntd.0004703 ; PubMed Central PMCID: PMCPMC4871561. 27192199 10.1371/journal.pntd.0004703 PMC4871561

[20] Barreto JG, Bisanzio D, Frade MA, Moraes TM, Gobbo AR, de Souza Guimaraes L, et al Spatial epidemiology and serologic cohorts increase the early detection of leprosy. BMC infectious diseases. 2015;15:527 Epub 2015/11/18. doi: 10.1186/s12879-015-1254-8 ; PubMed Central PMCID: PMCPMC4647818. 26573912 10.1186/s12879-015-1254-8 PMC4647818

[21] Groenen G, Pattyn SR, Ghys P, Tshilumba K, Kuykens L, Colston MJ. A longitudinal study of the incidence of leprosy in a hyperendemic area in Zaire, with special reference to PGL-antibody results. The Yalisombo Study Group. Int J Lepr Other Mycobact Dis. 1990;58(4):641–50. . 2280114

[22] Bagshawe AF, Garsia RJ, Baumgart K, Astbury L. IgM serum antibodies to phenolic glycolipid-I and clinical leprosy: two years' observation in a community with hyperendemic leprosy. Int J Lepr Other Mycobact Dis. 1990;58(1):25–30. Epub 1990/03/01. . 2181044

[23] Chanteau S, Glaziou P, Plichart C, Luquiaud P, Plichart R, Faucher JF, et al Low predictive value of PGL-I serology for the early diagnosis of leprosy in family contacts: results of a 10-year prospective field study in French Polynesia. Int J Lepr Other Mycobact Dis. 1993;61(4):533–41. Epub 1993/12/01. . 8151183

[24] Schuring RP, Moet FJ, Pahan D, Richardus JH, Oskam L. Association between anti-pGL-I IgM and clinical and demographic parameters in leprosy. Leprosy review. 2006;77(4):343–55. Epub 2007/03/09. . 17343221

[25] van Hooij A, Tjon Kon Fat EM, van den Eeden SJF, Wilson L, Batista da Silva M, Salgado CG, et al Field-friendly serological tests for determination of M. leprae-specific antibodies. Scientific reports. 2017;7(1):8868 Epub 2017/08/23. doi: 10.1038/s41598-017-07803-7 . 28827673 10.1038/s41598-017-07803-7 PMC5566372

[26] Corstjens PL, van Hooij A, Tjon Kon Fat EM, van den Eeden SJ, Wilson L, Geluk A. Field-Friendly Test for Monitoring Multiple Immune Response Markers during Onset and Treatment of Exacerbated Immunity in Leprosy. Clin Vaccine Immunol. 2016;23(6):515–9. Epub 2016/04/01. doi: 10.1128/CVI.00033-16 ; PubMed Central PMCID: PMCPMC4895013. 27030588 10.1128/CVI.00033-16 PMC4895013

[27] Bakker MI, May L, Hatta M, Kwenang A, Klatser PR, Oskam L, et al Genetic, household and spatial clustering of leprosy on an island in Indonesia: a population-based study. BMC medical genetics. 2005;6:40 Epub 2005/11/26. doi: 10.1186/1471-2350-6-40 ; PubMed Central PMCID: PMCPMC1318483. 16307680 10.1186/1471-2350-6-40 PMC1318483

[28] Hagge DA, Parajuli P, Kunwar CB, Rana D, Thapa R, Neupane KD, et al Opening a Can of Worms: Leprosy Reactions and Complicit Soil-Transmitted Helminths. EBioMedicine. 2017;23:119–24. 28882756 10.1016/j.ebiom.2017.08.026 PMC5605364

[29] Feenstra SG, Nahar Q, Pahan D, Oskam L, Richardus JH. Recent food shortage is associated with leprosy disease in Bangladesh: a case-control study. PLoS Negl Trop Dis. 2011;5(5):e1029 Epub 2011/05/17. doi: 10.1371/journal.pntd.0001029 ; PubMed Central PMCID: PMCPMC3091833. 21572979 10.1371/journal.pntd.0001029 PMC3091833

[30] Roset Bahmanyar E, Smith WC, Brennan P, Cummings R, Duthie M, Richardus JH, et al Leprosy Diagnostic Test Development As a Prerequisite Towards Elimination: Requirements from the User's Perspective. PLoS neglected tropical diseases. 2016;10(2):e0004331 Epub 2016/02/13. doi: 10.1371/journal.pntd.0004331 ; PubMed Central PMCID: PMCPMC4750857. 26866699 10.1371/journal.pntd.0004331 PMC4750857

[31] Barth-Jaeggi T, Steinmann P, Mieras L, van Brakel W, Richardus JH, Tiwari A, et al Leprosy Post-Exposure Prophylaxis (LPEP) programme: study protocol for evaluating the feasibility and impact on case detection rates of contact tracing and single dose rifampicin. BMJ Open. 2016;6(11):e013633 Epub 2016/11/20. doi: 10.1136/bmjopen-2016-013633 ; PubMed Central PMCID: PMCPMC5128948. 27856484 10.1136/bmjopen-2016-013633 PMC5128948

