# Supplementary material for: Longitudinal assessment of anti-PGL-I serology in contacts of leprosy patients in Bangladesh
Source: PLoS Negl Trop Dis. 2017 Dec 11;11(12):e0006083. doi: 10.1371/journal.pntd.0006083 (PMC5746281; doi:10.1371/journal.pntd.0006083)
Supplement: S1 Checklist — (DOCX) [file pntd.0006083.s001.docx]

STROBE Statement—checklist of items that should be included in reports of observational studies

|  | Item No. | Recommendation | Page  No. | Relevant text from manuscript |
| --- | --- | --- | --- | --- |
| **Title and abstract** | 1 | (*a*) Indicate the study’s design with a commonly used term in the title or the abstract | 1, 2 | Longitudinal assessment, field-trial |
|  |  | (*b*) Provide in the abstract an informative and balanced summary of what was done and what was found | 2 | Please read the abstract |
| Introduction | | | |  |
| Background/rationale | 2 | Explain the scientific background and rationale for the investigation being reported | 4,5 | Please read the introduction |
| Objectives | 3 | State specific objectives, including any prespecified hypotheses | 5 | Please read the last paragraph of the introduction |
| Methods | | | |  |
| Study design | 4 | Present key elements of study design early in the paper | 6 | Please see the section ‘study participants’ of the methods, we refer to the COLEP study |
| Setting | 5 | Describe the setting, locations, and relevant dates, including periods of recruitment, exposure, follow-up, and data collection | 6 | Please see the section ‘study participants’ of the methods |
| Participants | 6 | (*a*) *Cohort study*—Give the eligibility criteria, and the sources and methods of selection of participants. Describe methods of follow-up  *Case-control study*—Give the eligibility criteria, and the sources and methods of case ascertainment and control selection. Give the rationale for the choice of cases and controls  *Cross-sectional study*—Give the eligibility criteria, and the sources and methods of selection of participants | 6 | Please see the section ‘test group selection’ of the methods |
|  |  | (*b*) *Cohort study*—For matched studies, give matching criteria and number of exposed and unexposed  *Case-control study*—For matched studies, give matching criteria and the number of controls per case |  |  |
| Variables | 7 | Clearly define all outcomes, exposures, predictors, potential confounders, and effect modifiers. Give diagnostic criteria, if applicable | 6,7,8 | Please see the Methods section |
| Data sources/ measurement | 8* | For each variable of interest, give sources of data and details of methods of assessment (measurement). Describe comparability of assessment methods if there is more than one group | 7,8 | Please see the section ‘synthetic PGL-I up to Statistical Analyses’ of the methods |
| Bias | 9 | Describe any efforts to address potential sources of bias |  | Not applicable |
| Study size | 10 | Explain how the study size was arrived at | 6 | Please see the section ‘test group selection’ of the methods |

Continued on next page

| Quantitative variables | 11 | Explain how quantitative variables were handled in the analyses. If applicable, describe which groupings were chosen and why | 6,7,8 | Please see the section ‘test group selection up to Statistical Analyses’ of the methods |
| --- | --- | --- | --- | --- |
| Statistical methods | 12 | (*a*) Describe all statistical methods, including those used to control for confounding | 8 | Please read ‘statistical analyses’ in the methods section |
|  |  | (*b*) Describe any methods used to examine subgroups and interactions |  | Not applicable |
|  |  | (*c*) Explain how missing data were addressed | 6 | Please read the section ‘test group selection’ in the methods section (i.e. missing data was not included in analyses) |
|  |  | (*d*) *Cohort study*—If applicable, explain how loss to follow-up was addressed  *Case-control study*—If applicable, explain how matching of cases and controls was addressed  *Cross-sectional study*—If applicable, describe analytical methods taking account of sampling strategy | 6 | Please read the section ‘test group selection’ in the methods section |
|  |  | (*e*) Describe any sensitivity analyses |  | Not applicable |
| Results | | | | |
| Participants | 13* | (a) Report numbers of individuals at each stage of study—eg numbers potentially eligible, examined for eligibility, confirmed eligible, included in the study, completing follow-up, and analysed | 9 | Please read the Results section line 2-9. |
|  |  | (b) Give reasons for non-participation at each stage | 9 | Please read the Results section line 2-9. |
|  |  | (c) Consider use of a flow diagram |  | Not applicable |
| Descriptive data | 14* | (a) Give characteristics of study participants (eg demographic, clinical, social) and information on exposures and potential confounders | 9 | Please read the Results section line 7-14 |
|  |  | (b) Indicate number of participants with missing data for each variable of interest | 9,10 | Please read the Results section. |
|  |  | (c) *Cohort study*—Summarise follow-up time (eg, average and total amount) | 9,10 | Please read the Results section |
| Outcome data | 15* | *Cohort study*—Report numbers of outcome events or summary measures over time | 9 | Please read the Results section line 7-14 |
|  |  | *Case-control study—*Report numbers in each exposure category, or summary measures of exposure |  |  |
|  |  | *Cross-sectional study—*Report numbers of outcome events or summary measures |  |  |
| Main results | 16 | (*a*) Give unadjusted estimates and, if applicable, confounder-adjusted estimates and their precision (eg, 95% confidence interval). Make clear which confounders were adjusted for and why they were included | 9,10 | Please read the Results section |
|  |  | (*b*) Report category boundaries when continuous variables were categorized | 9 | “a positive anti-PGL-I Ab level of >0,150” |
|  |  | (*c*) If relevant, consider translating estimates of relative risk into absolute risk for a meaningful time period |  | Not applicable |

Continued on next page

| Other analyses | 17 | Report other analyses done—eg analyses of subgroups and interactions, and sensitivity analyses |  | Not applicable |
| --- | --- | --- | --- | --- |
| Discussion | | | | |
| Key results | 18 | Summarise key results with reference to study objectives | 11-15 | Please read the Discussion |
| Limitations | 19 | Discuss limitations of the study, taking into account sources of potential bias or imprecision. Discuss both direction and magnitude of any potential bias | 11-15 | Please read the Discussion |
| Interpretation | 20 | Give a cautious overall interpretation of results considering objectives, limitations, multiplicity of analyses, results from similar studies, and other relevant evidence | 11-15 | Please read the Discussion |
| Generalisability | 21 | Discuss the generalisability (external validity) of the study results | 11-15 | Please read the Discussion |
| Other information | |  | | |
| Funding | 22 | Give the source of funding and the role of the funders for the present study and, if applicable, for the original study on which the present article is based |  | This information was provided when the article was submitted |

*Give information separately for cases and controls in case-control studies and, if applicable, for exposed and unexposed groups in cohort and cross-sectional studies.

**Note:** An Explanation and Elaboration article discusses each checklist item and gives methodological background and published examples of transparent reporting. The STROBE checklist is best used in conjunction with this article (freely available on the Web sites of PLoS Medicine at http://www.plosmedicine.org/, Annals of Internal Medicine at http://www.annals.org/, and Epidemiology at http://www.epidem.com/). Information on the STROBE Initiative is available at www.strobe-statement.org.
